# An Exploration of Social Circles and Prescription Drug Abuse Through Twitter

**DOI:** 10.2196/jmir.2741

**Published:** 2013-09-06

**Authors:** Carl Lee Hanson, Ben Cannon, Scott Burton, Christophe Giraud-Carrier

**Affiliations:** ^1^Computational Health Science Research GroupDepartment of Health ScienceBrigham Young UniversityProvo, UTUnited States; ^2^Computational Health Science Research GroupDepartment of Computer ScienceBrigham Young UniversityProvo, UTUnited States

**Keywords:** prescription drug abuse, social media, social circles, Twitter

## Abstract

**Background:**

Prescription drug abuse has become a major public health problem. Relationships and social context are important contributing factors. Social media provides online channels for people to build relationships that may influence attitudes and behaviors.

**Objective:**

To determine whether people who show signs of prescription drug abuse connect online with others who reinforce this behavior, and to observe the conversation and engagement of these networks with regard to prescription drug abuse.

**Methods:**

Twitter statuses mentioning prescription drugs were collected from November 2011 to November 2012. From this set, 25 Twitter users were selected who discussed topics indicative of prescription drug abuse. Social circles of 100 people were discovered around each of these Twitter users; the tweets of the Twitter users in these networks were collected and analyzed according to prescription drug abuse discussion and interaction with other users about the topic.

**Results:**

From November 2011 to November 2012, 3,389,771 mentions of prescription drug terms were observed. For the 25 social circles (n=100 for each circle), on average 53.96% (SD 24.3) of the Twitter users used prescription drug terms at least once in their posts, and 37.76% (SD 20.8) mentioned another Twitter user by name in a post with a prescription drug term. Strong correlation was found between the kinds of drugs mentioned by the index user and his or her network (mean *r*=0.73), and between the amount of interaction about prescription drugs and a level of abusiveness shown by the network (*r*=0.85, *P*<.001).

**Conclusions:**

Twitter users who discuss prescription drug abuse online are surrounded by others who also discuss it—potentially reinforcing a negative behavior and social norm.

## Introduction

### Prescription Drug Abuse

Prescription drug abuse has become the fastest-growing drug problem in the United States [1], contributing to approximately 27,000 overdose deaths during 2007 [2]. Nearly one-third of individuals over the age of 12 who were first-time drug users in 2009 started by abusing a nonmedical prescription drug [3]. It is estimated that 48 million Americans (approximately 20% of the population) aged 12 and older have used prescription drugs for nonmedical reasons at some point in their lifetime [4].

Even though death only occurs in the most severe cases of abuse, the negative health consequences of prescription drug abuse are many, ranging from simple drowsiness and nausea to lack of coordination, disorientation, paranoia, and seizures. A recent study also found that there may be an emerging trend of (ab)using prescription drugs among adolescents to facilitate unwanted sexual contact [5]. A teen addiction treatment center in Iowa similarly warns against unwanted sexual behavior as one of the consequences of prescription drug abuse [6]. While there does not seem to be evidence of more at-risk sexual behaviors, such as sex-for-drugs, since most people have easy access to prescription drugs either from friends and relatives or through “doctor shopping”, this trend still raises concerns about the limited, yet real, danger of prescription drug abuse increasing exposure to and spread of HIV.

The Office of National Drug Control Policy (ONDCP) Prescription Drug Abuse Prevention Plan includes four major areas of focus: education, monitoring, proper medication disposal, and enforcement [7]. Current public health intervention strategies are largely aimed at prescribers and distributors. In many states, doctors receive training on how to identify abusers and patients that doctor shop. In some states, pharmacies and distributors are required to report the amount of controlled substances disbursed each week. While these measures have proven to reduce rates of overdose and overdose deaths, primary preventative measures among end users of prescription drugs have not been explored or implemented as widely. The inherent difficulty of identifying abusers and redirectors of prescription drugs fosters an easy environment for abuse without real threat of legal repercussion.

### Social Networks and Social Media

Relationships embedded in one’s social network are an important influencing factor and contributor to health behavior and outcome, even beyond individual attributes such as age, sex, education level, income, and occupation [8-12]. In the context of prescription drug abuse, a recent study of the co-usage network of a population of 503 prescription drug abusers in rural Appalachian areas shows that daily OxyContin use is significantly associated with higher effective size of ego networks (a measure of social capital), and thus “speak to the importance of peer networks in determining social capital and social norms, which has vast implications for intervention research” [13]. It has been found that people, including youth, often learn to abuse prescription drugs by observing a family member, or other members of their social network, model the abuse of prescription drugs [14,15]. Within families, the practice of “friendly sharing” of prescription drugs has become commonplace [16]. Recent research has also identified social groups or informal economic markets where drug transactions can occur. An established market for prescription drug distribution has been identified in junior high and high school classes. Among students in Nova Scotia who had been prescribed stimulants, about 22% reported giving away or selling their medications, while another 7.3% experienced theft or were forced into giving away their prescriptions [17].

Research has revealed that the Internet provides ready access to drugs, including prescription medications [16,18]. More recently, evidence also suggests that participation in social media sites may increase one’s risk of substance abuse, especially among adolescents. The National Center on Addiction and Substance Abuse began collecting data to explore the influence of social networking and substance abuse in 2011. Their findings reveal that teens who spend time social networking online are five times more likely to use tobacco, three times more likely to use alcohol, and two times more likely to use marijuana [16].

While studies have demonstrated the influence of social relationships on prescription drug abuse in the real world as well as ready access to the drugs, little is known about these influences in cyberspace. Social media applications, such as Twitter, offer a way to observe the conversations of individuals and their social circles directly, providing a mechanism to monitor end users of prescription drugs. By monitoring individual conversations, studies have demonstrated the validity of identifying health topics on Twitter [19,20], including prescription drug misuse [21]. In addition, social media applications are platforms for networking and as such are rich with relationships. These relationships make up important social circles that have the capacity to influence behavior due to unique norms and values of the group. Indeed, no social media user is an island, and the *social* element of social media has particular relevance in public health research.

Infodemiology represents a new field of study where the Internet, even social media platforms, provides channels through which to explore the distribution and determinants of information [22,23]. A growing body of research demonstrates the validity of this methodology for understanding public health challenges [21,24-31]. This study extends previous health research in social media by analyzing not only the content of social media posts, but also the relationships among users. Specifically, online social circles of prescription drug abusers are identified with discussions and interactions of these networks are analyzed. Few studies have explored the influence of online relationships on alcohol and other drug use [32,33]. To the best of our knowledge, this is the first work to focus on the relational component of these networks through social media, with regard to prescription drug abuse.

The purpose of this study was to investigate the social circles of prescription drug abusers on Twitter and to observe the discussion and engagement of these users regarding prescription drug abuse. To fulfill the purpose of this study, the following hypotheses were explored:

H1: People discuss prescription drug abuse on Twitter.

H2: People who discuss prescription drug abuse on Twitter belong to social circles that engage with each other about prescription drug abuse.

H3: Social engagement about prescription drug abuse varies across social circles of those who discuss it, and higher engagement correlates with higher levels of abuse.

## Methods

### Overview

A distinction exists between prescription drug abuse and prescription drug misuse. The former refers to using a drug with the intent of deriving some side effect, usually of a euphoric nature (ie, getting high). The latter refers to increasing dosage in an attempt to improve the drug efficacy or to sharing the drug with someone whose symptoms may call for it but to whom the drug has not been prescribed. Either way, one can easily argue that “no matter the intention of the person...taking a drug other than the way it is prescribed can lead to dangerous outcomes that the person may not anticipate” (page 1) [34]. Hence, throughout the paper, any improper use and user are referred to simply as abuse and abuser, respectively.

To evaluate the discussion of prescription drug abuse among social media users, Twitter users mentioning prescription drugs were identified, and their tweets as well as those of their network were analyzed.

### Study Setting

Social media applications such as Twitter provide channels for social networking with others who may have similar interests and needs. Twitter provides users with a platform to share short messages (“tweets”) among themselves. Twitter users can “follow” others to subscribe to a feed of tweets from users of interest; they can also broadcast their messages to all of their followers or direct messages at specific users (“mentions”). By default, tweets are public; hence, it is generally possible for a user, X, to see the tweets of a user, Y, even though X may not be following Y or Y did not mention X explicitly. Because Twitter users tend to post messages as events occur in their lives, tweets are an ideal source for researchers to observe natural and timely interactions among people. As such, Twitter was used to observe discussion and engagement with regards to prescription drug abuse.

This study was approved by the institutional review board at Brigham Young University, Provo, Utah.

### Identifying Users and Networks

Twitter provides an application programming interface (API) that enables programmatic consumption of the content and the relationships of its tweets and users. The Twitter Streaming API provides a means of obtaining tweets as they occur, filtered by specific criteria, such as a list of keywords. The Twitter API also enables discovering the people following and followed by a given user, as well as retrieving up to 3200 of a user’s most recent tweets.

To identify a set of tweets mentioning prescription drugs, the Twitter stream was filtered for prescription drug terms, producing a set of all tweets mentioning these terms from November 29, 2011 through November 14, 2012. From this set, potential prescription drug abusers were identified for analysis along with their networks. In order to select those Twitter users who had some discussion of prescription drugs, but that were still regular users, Twitter users that mentioned prescription drugs in at least 10 tweets but less than 100 were selected at random. Evaluation revealed that Twitter users in this range were most likely regular users as opposed to accounts devoted to online drug sales, automated feeds, and companies, which tended to tweet more frequently about prescription drugs. A sample of 25 networks was obtained for further analysis. In order to select the 25 networks, a member of the research team sampled networks and read through prescription drug tweets to verify evidence of prescription drug abuse based on a pattern of prescription drug tweets that matched one or more of the categories of abuse. Networks were excluded from the sample if prescription drug tweets did not match any of the categories of abuse. Likely prescription drug abusers tended to have tweets that matched the categories of abuse. For example, one of the 25 index users was selected because he/she had a pattern of tweeting about Adderall and Xanax (45 and 34 tweets respectively) and 26 of those tweets matched several of the abuse categories. Most alarming was that 11 of the abuse tweets were about co-ingestion. One of these co-ingestion tweets stated, “Adderall + Benadryl has put me in a weird awake/tired haze. Relatively certain that I’m saying things i wont [sic] remember in the morning”.

The social circles of each of the 25 index Twitter users were discovered. Unlike a traditional ego network that consists of all the individuals ego has a direct connection to, a social circle is a densely connected set of mutually aware individuals that surround ego, where some may be included in the circle by virtue of their many connections to ego’s alters. Social circles capture the intuition that someone who influences ego’s alters may exert a stronger influence on ego, though indirectly, than some of ego’s alters. Finding a social circle around one or a small group of individuals is an instance of the community search problem [35], a query-based version of the traditional community mining problem [36]. In the context of Twitter, however, there are two additional constraints: (1) the Twitter graph cannot be feasibly known, and (2) the “follow” relation in Twitter is directed. As a result, a local social circle discovery algorithm designed specifically for directed graphs must be used [37].

**Figure 1:**
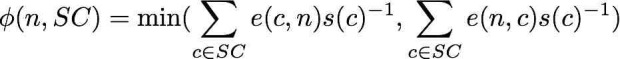
Local social circle discovery measure.

Intuitively, the algorithm initializes the social circle with the index Twitter user and then iteratively adds new members to the social circle until a prespecified size has been reached. At each step, the algorithm considers all Twitter users followed by at least one member of the current social circle, and selects the one with the highest score. The score of each candidate is the minimum of the number of individuals in the social circle it links to and the number of individuals it is linked from. To ensure that new members do not cause the social circle to drift away from the initial Twitter user, the value of a connection to a social circle member is discounted according to the step at which that member was added to the circle. Figure 1 shows the score of a candidate node *n* with respect to the social circle *SC* [37], where *e(x, y)* is an edge indicator function (ie, *e(x, y)* = 1 if there is an edge from *x* to *y* and 0 otherwise), and *s(c)* is the step in which node *c* was added to the social circle.

**Figure 2:**
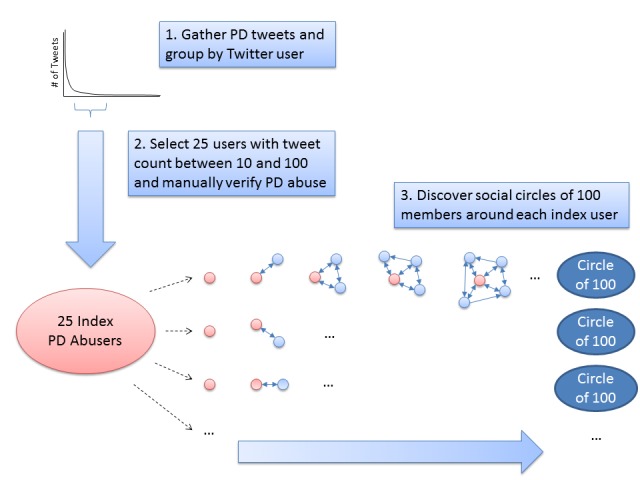
Social circle discovery process.

To increase the cohesiveness of the social circle, every 5 iterations, the Twitter user with the lowest score is removed from the social circle. Upon completion, the algorithm returns a social circle composed of dense connections of mutually aware nodes that surround the index Twitter user. Note that, in general, individuals belong to different social circles that may best be specified by including additional people in the query set (eg, work colleagues would likely produce a professional social circle, relatives would likely produce a family social circle). Here, however, the index Twitter user is used as the sole query node to avoid biasing the algorithm toward any specific social circle, and instead simply discovering the most natural dense set surrounding that individual. The process of identifying these social circles and users is illustrated in Figure 2.

To ensure consistent comparison across networks, a social circle of the same size was discovered around each index Twitter user. After each social circle was identified, the most recent tweets of each Twitter user in the social circle (up to 3200 per user, the maximum allowed by the Twitter API) were obtained for content analysis. The size of social circles was set to 100 since there are significant computational costs associated with extracting this content, most social media users effectively maintain only between 100 and 200 friends [38], and the closeness of friendship tends to decline as social circles grow.

### Content Categorization

Once a social circle and its corresponding tweets were obtained, tweets were categorized by mention of a particular substance, and further categorized by the manner in which that substance was mentioned. Table 1 lists the drug categories and the filter terms used to categorize the tweet. For example, a tweet was categorized as mentioning painkillers if it contained terms such as “painkiller”, “oxycontin”, or “lortab”.

Tweets matching the drugs in Table 1 were further categorized into 8 different types of abusive or risk behaviors defined in Table 2: taking larger doses (overdose), co-ingestion, taking more frequent doses, alternative motives (dependence or need the drug due to addiction), alternative routes of admission, legitimacy of obtaining, redistributing (trading/selling), and seeking [39].

Tweets that matched the drugs in Table 1 were also further analyzed to determine if they contained “mentions” to other Twitter users (where an author references another user by the *@username* convention). Social network graphs were then constructed to show such connections among Twitter users. The graphs are directed and weighted. The weight of an edge is defined by the number of tweets from one user to another that included prescription drug terms.

**Table 1:** Keywords for prescription drugs.

Drugs	Keywords^a^
Adderall	adderall
Xanax	xanax
Klonopin	klonopin
Valium	valium; sleeping pills
Painkillers	painkiller*; pain killer*; narcotic painkiller*; oxycontin; vicodin; percodan; percocet; darvon; lortab; lorcet; dilaudid; demerol; lomotil; kadian; avinza; codeine; duragesic; methadone
Depressants	mebaral; nembutal; sodium pentobarbital; halcion; prosam; ativan; librium; depressant*
Stimulants	dexedrine; ritalin; concerta; amphetamines; stimulant*

^a^The “*” matches 0 or more additional characters.

**Table 2:** Keywords for risk/abusive behaviors.

Risk/Abusive Behaviors	Keywords^a^
Larger doses/overdose	too many; two; three; double; too much; overdose; crash; strong enough; max; too many
Co-ingestion^b^	alcohol; coffee; white; red; wine; vodka; shots; patron; booze; margarita; mimosa; xanax; painkiller; caffeine; alcohol; happy pills; adderall; concerta; cocaine; rum
More frequent doses	enough; pop; popping; not enough; another; enough; pop*
Alternative motives/dependence^c^	test; final; study; studying; problems; college; class; breakfast; rely; sleep; sleeping; work; family problems; sleep*; stress*; stressful; stress; skinny
Alternative routes of admission	snort; crush; inject; snort; inhale
Legitimacy of obtaining	steal*
Trading/selling	buy; sell; trade; share; spend; buy; bring
Seeking	need; want; needing; wanting; wish; need

^a^The “*” matches 0 or more additional characters.

^b^Co-ingestion keywords for xanax and adderall did not include the keywords “xanax” and “adderall” respectively.

^c^The keywords “test”, “final”, “study”, and “studying” were exclusively used as keywords for Adderall. “Skinny” was exclusive to Stimulants.

## Results

The tweets collected during the study period contained 3,389,771 references to prescription drug terms. Table 3 shows the number of co-occurrences of these references with one of the categories defined by the terms in Table 2. The large number of references to alternative motives was due primarily to discussion of Valium as a sleep aid.

The 25 social circles discovered around the 25 index Twitter users gave rise to a total of 2227 unique Twitter users, 7290 prescription drug tweets, and 2788 directed prescription drug tweets. Statistics of these social circles are shown in Table 4. As shown, the social circles range from 14% to 87% (mean 53.96%, SD 24.8) of the Twitter users in the social circle tweeting about prescription drugs at least once.

Index users and their social circles typically tweeted about similar drugs. For each index Twitter user, a topic vector was determined according to the proportion of his or her prescription drug tweets that matched each of our prescription drug categories, and a topic vector was also created for the aggregated tweets of the rest of the social circle. The topic vectors of index Twitter users were correlated with those of their social circle, and Pearson’s correlation coefficients ranged from -0.14 to 0.99 (mean 0.73, SD 0.31). The mean of these correlation coefficients was computed by first applying Fisher’s *z* transformation.

Using the abusive behaviors content categories of Table 2, each of the tweets of the index Twitter users and their social circles were categorized according to potential abuse. Although not a perfect metric for abuse, the number of abuse categories a Twitter user mentions is used as surrogate for a level of abuse. Thus, a Twitter user who has tweets matching four of the abuse categories is considered to be at a higher level than a Twitter user who only has tweets from one of them. As shown in Table 4, the mean number of the people in the social circle with tweets matching at least one abuse category was 33.2 (SD 18.8), and 16.8 (SD 10.9) users had tweets matching at least two. The level of abuse is strongly correlated with the number of Twitter users interacting with others about prescription drugs. Comparing the percentage of the social circle that interacts about prescription drugs to the percentage that matched at least one abuse category yields a Pearson’s correlation coefficient of *r*=0.85 (*P*<.001), and comparing against those who matched two or more abuse categories, *r*=0.81 (*P*<.001).

**Figure 3:**
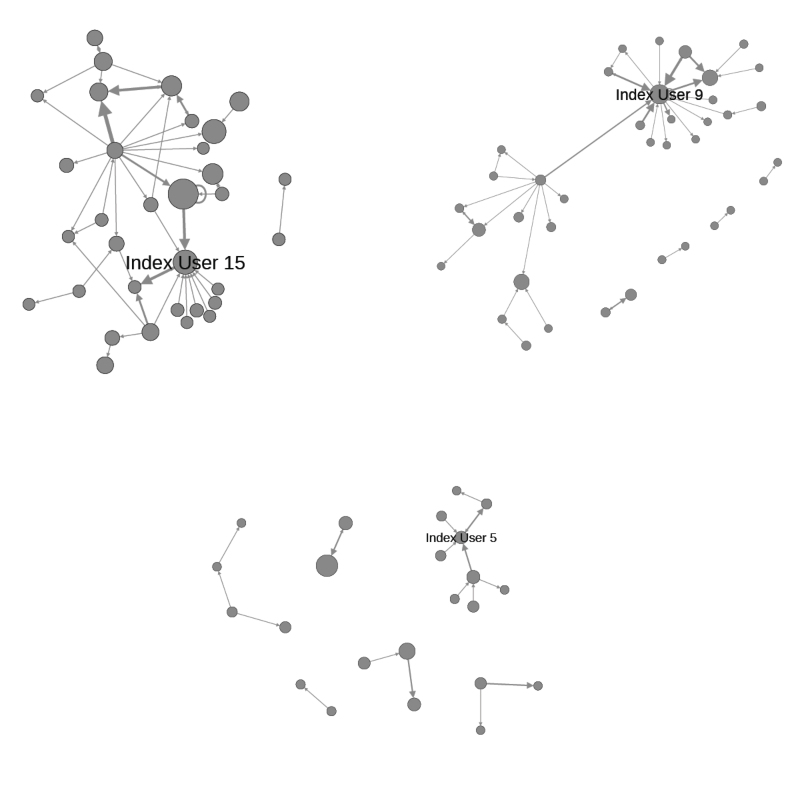
Prescription drug interaction graphs.

In addition to the quantitative evaluation of these interactions, interesting patterns can also be observed through visual inspection of the graphs of interactions among Twitter users in each social circle. Figure 3 shows three graphs, where the nodes represent users, and the edges indicate that the source user mentioned the destination user along with a prescription drug term. The weight of the edges (as shown by the thickness of the line) denotes the number of mentions. The size of the nodes represents the number of prescription drug tweets.

**Table 3:** Number of prescription drug tweets by drug category.

Category	Adderall	Xanax	Klonopin	Valium	Painkillers	Depress	Stim	Total
Drug total	412,314	486,670	58,527	917,805	1,215,574	17,364	281,517	3,389,771
Larger doses / overdose	11,397	9508	880	22,263	28,186	218	2085	74,537
Co-ingestion	44,179	24,794	5411	47,657	34,178	1027	3181	160,427
More frequent doses	10,636	18,070	567	15,808	22,764	107	2566	70,518
Alternative motives / dependence	39,459	18,664	105	617,672	38,135	806	1868	716,709
Alternative routes of admission	1316	1657	73	701	1641	17	265	5670
Legitimacy of obtaining	363	400	16	339	1032	6	117	2273
Trading / selling	20,941	63,763	17,000	65,926	95,962	4913	2873	271,378
Seeking	46,138	52,852	2069	165,955	63,165	675	8808	339,662

**Table 4:** Summary statistics for prescription drug tweets within social circles.

Network	Prescription drug Tweets		Prescription drug Tweet mentions^d^		Topic correlation	One or more abuse categories^e^	Two or more abuse categories
	n	n	n	n	*r*	n	n
	Tweets^f^	Users^g^	Tweets^h^	Users^i^		Users^j^	Users
1	136	48	55	32	0.28	25	9
2	99	28	22	12	0.26	13	1
3	67	14	26	11	0.06	8	2
4	508	84	290	72	0.59	38	18
5	352	46	97	34	0.69	34	22
6	258	72	37	29	0.92^a^	27	12
7	311	69	40	27	0.76^b^	39	18
8	52	17	14	9	0.1	8	6
9	553	61	142	40	0.83^b^	33	18
10	359	76	156	51	0.89^a^	58	21
11	159	32	73	26	0.72	18	11
12	449	77	300	71	-0.14	36	18
13	446	87	302	84	0.74	73	39
14	378	79	112	42	0.65	55	30
15	629	61	140	42	0.99^a^	34	21
16	75	31	36	23	0.82^b^	28	11
17	512	84	244	64	0.93^a^	58	33
18	91	25	35	20	0.89^a^	9	3
19	75	30	28	17	0.37	17	8
20	75	20	24	16	0.77^b^	10	5
21	143	46	80	36	0.3	25	11
22	512	79	91	48	0.86^b^	54	35
23	417	69	142	47	0.6	52	28
24	387	83	249	70	0.97^a^	60	30
25	247	31	53	21	0.61	19	10
Mean	291.6	53.9	111.5	37.8	0.73^c^	33.2	16.8
SD	183.5	24.8	94.9	21.2	0.31	18.8	10.9

^a^ *P*<.01

^b^ *P*<.05

^c^The mean of topic correlation coefficients was computed using Fisher’s z transformation.

^d^Mentions refers to tweets directed at another user.

^e^Tweets matching abuse categories.

^f^Total prescription drug tweets from the social circle

^g^Number of people in social circle that produced tweets in column two

^h^Mention tweets (Subset of column two)

^i^Number of people in social circle that produced tweets in column four

^j^Number of people in social circle that had tweets classified into one or more abuse categories

In addition to simply talking about prescription drugs, Twitter users in these social circles also interact with each other about the topic, using the *@username* convention. Examples of mention tweets from the sample include, “@*** Haha! For me it's a nice ritalin/sangria combo :)”, “RT @*** I should win a lifetime achievement award...I've been taking Xanax for years without overdosing.”, and “@*** lol thanks....but im [sic] pretty emotionally stable. It's called being in a Xanax haze”. As shown in Table 4, the networks range from 9 to 84 (mean 37.8, SD 21.2) Twitter users in the social circle (n=100) interacting with another Twitter user about prescription drugs at least once.

## Discussion

### Principal Findings

The purpose of this study was to explore the online social circles of prescription drug abusers to observe the discussion and engagement of these Twitter users regarding prescription drug abuse. Findings revealed that for the identified prescription drug abusers on Twitter, their social circles consisted of other Twitter users who also discussed abuse of prescription drugs.

The study was guided by three research hypotheses. As shown in Table 3, significant discussion of prescription drug abuse was observed on Twitter (hypothesis 1). These findings are consistent with previous research exploring prescription drug abuse through Twitter [21]; however, the current study explores multiple prescription drug mentions beyond just Adderall. While not all of these tweets are necessarily in reference to abuse, those matching the abuse categories defined in Table 2 are very likely to be discussion of abuse of prescribed substances. Even if not all of these references denote actual behavior on the part of individuals, the simple act of discussing the behavior within a social circle can impact the social norms of those within that circle.

Those who are not engaged in prescription drug abuse are still being exposed to others’ tweets concerning the matter. They may not be participating in the conversation, but they are observing the sentiment and potentially forming ideas and norms about the abuse of prescription drugs. While actual drug abuse remains mostly a private affair, it seems to be discussed in a very open manner online for all to observe. It may be that abusers are now, through social media, finding support for their abuse and feel a sense of safety in opening up to others. Uses and gratification theory suggests that individuals make decisions about their media choice based on the extent to which that media gratifies a communication need [40]. Duffy and Thorson [41] expand this idea in their Health Communication Media Choice Model and suggest that connectivity is an important need that can be fulfilled through social media. They define connectivity as the “need to relate, support, engage with, and communicate with others face-to-face through media” (page 102) [41]. Social media facilitates the connectivity process by allowing people to engage with and observe others’ sentiment on a given subject. Regardless of a person’s openness about their behavior, prescription drugs are being discussed on Twitter and many are being exposed to tweets and conversations of an abusive nature through their social circles.

As shown in Table 4, there is a significant amount of discussion about prescription drugs in the social circles of the index Twitter users, with a mean of 53.9 (SD 24.8) users in the social circles posting about a topic at least once, and an mean of 291.6 (SD 183.5) tweets per social circle (hypothesis 2). In addition, the high correlation between the substances discussed by the index Twitter user and his or her social network, shows that these users are engaged in discussions with others of like minds. These findings confirm our hypothesis and also show consistency with the offline world about the social context of prescription drug abuse [14,15,17,42].

It is not clear whether index Twitter users developed their behavior from exposure to their online social circle, or whether they sought out the company of others supportive of their viewpoints. But it is clear that each of these Twitter users is in an environment that potentially supports their behavior. This may have interesting ramifications, because these users may not be in close proximity to one another physically, and yet they may find reinforcement for their attitudes from their online connections. Thus, while prescription drug abusers may not feel comfortable sharing their experiences with their physical neighbors, who might not approve of abusive behavior, they can develop online associations with those that do. These findings are consistent with recent research exploring the impact of online social circles on young adult alcohol use [32,33].

In addition to knowing that Twitter users are talking about prescription drugs, it is also relevant to discover if they are also talking *to each other* about prescription drugs (hypothesis 3). When Twitter users mention one another by their username (using the *@username* convention), these tweets are aggregated into a separate list in the interface, and can also produce other alerts (eg, email) raising the user’s level of awareness of the tweet. In addition, the author may be directly soliciting a response from the user. Thus, the analysis of the number of tweets that discuss prescription drugs and also mention a specific user provides a quantified measure to observe engagement among these users about the topic.

The fact that, on average, 37.8 (SD 21.2) Twitter users in a social circle interact with another user at least once shows that there is indeed a significant level of engagement in addition to simply talking about the topic. Furthermore, hypothesis 3 is confirmed by the fact that the percentage of social circles interacting about prescription drugs correlates so strongly with the percentage of social circles having tweets that match risk/abusive behavior categories (*r*=0.85 for one category and *r*=0.81 for two categories). Social engagement can also be observed through the interaction graphs shown in Figure 3. It is interesting to observe how some users who discuss prescription drugs relatively frequently (as denoted by the larger size of the node), in many cases also have a large in-degree, showing that many others mention them in connection with prescription drugs.

With the rise of prescription drug abuse and its inherent danger, understanding the behaviors of abusers will be vital for public health professionals and prescribers in preventing overdose deaths and the blatant redirecting of the drugs. Many states are implementing prescription drug registries in response to the epidemic of abuse. These registries require prescribers and providers to report the distribution of controlled substances. While these registries can identify patients going to multiple doctors for the same medication, they do not address the growing problem of prescription drug redirection. This drug aftermarket is only facilitated by social media platforms like Twitter. The categorization keywords used in this study were able to identify users seeking, trading, and buying prescription drugs. For example, several seeking statements included, “Seriously. Need adderall. Will pay $$$. Help me.” and “looking to buy ~20-40 mg adderall, email ***”. While a drug registry may identify and limit an abuser in one state, that abuser can simply source drugs online from others in states where drug registries are not used and abusers are able to obtain excessive amounts of a drug. Another key risk behavior that drug registries cannot address is that of co-ingestion and nonmedical use. Co-ingestion is one of the deadliest drug abuse behaviors and a leading cause of overdose death.

Findings from this study have important implications for those professionals involved in the prevention and treatment of prescription drug abuse. Results indicate that Twitter is used as a platform for discussion about prescription drug abuse within social circles. As such, Twitter provides an additional “access point” to groups of individuals who are abusing prescription drugs. Innovative approaches to reaching these social circles might include online peer health advisors who have been trained to identify prescription drug abuse and appropriately intervene. In addition, enacting federal legislation meant to address the promotion of nonmedical use of prescription drugs through social media may help provide a safer online environment that is more supportive of healthy decision making, especially for adolescents who are most at risk [43].

### Limitations

Results from this study should be interpreted in light of the following limitations. First, while a keyword-based approach for identifying and categorizing tweets may exclude misspellings of the term, it does result in a highly precise set for analysis and, at a minimum, provides a lower bound for the amount of discussion. Second, through social media it is possible to observe only discussion, not actual behavior. Yet, as these are natural conversations among friends where people post about events that occur in their lives, there is no a priori reason to believe that on the whole people are falsifying their posts to portray events or behaviors that do not occur. Third, we may have underestimated the number of prescription drug abuse tweets. It is possible that there are other prescription drug abuse-related tweets that we missed because they were not covered by our keywords. It is also possible that not all tweets were delivered to us by the Twitter interface, although that is hard to know for certain. Also, tweets containing abuse-related keywords may not always refer to discussion of abuse. Last, this analysis was restricted to publicly available tweets, and as noted, it is possible that private tweets may in fact be more biased toward prescription drug abuse. Despite these limitations, it is likely that the general trends observed would not be affected.

### Conclusions

Understanding the prevalence of a problem or issue through social media is a good place to start; however, prevalence data fails to take advantage of the key aspect of social media: social networks and relationships. This work extends previous work by examining the social context of those discussing an important public health topic. While a major focus of this work has been about the reinforcement of negative behavior, the analysis of the interactions between people can provide insights into the normative aspects of social media. Whereas Twitter is a social media platform used to discuss and reinforce prescription drug abuse, prevention specialists should be mindful of this communication channel as another setting for understanding and monitoring prescription drug abuse and potentially intervening online.

## Abbreviations

API: application programming interface
ONDCP: Office of National Drug Control Policy
